# Dynamic Flux Balance Analysis to Evaluate the Strain Production Performance on Shikimic Acid Production in *Escherichia coli*

**DOI:** 10.3390/metabo10050198

**Published:** 2020-05-15

**Authors:** Yuki Kuriya, Michihiro Araki

**Affiliations:** 1 Graduate School of Medicine, Kyoto University, 54 ShogoinKawahara-cho, Sakyo-ku, Kyoto 606-8507, Japan; kuriya.yuki.7c@kyoto-u.ac.jp; 2 National Institutes of Biomedical Innovation, Health and Nutrition, 1-23-1 Toyama, Shinjuku-ku, Tokyo 162-8636, Japan; 3 Graduate School of Science, Technology and Innovation, Kobe University, 1-1 Rokkodai, Nada, Kobe, Hyogo 657-8501, Japan

**Keywords:** genome-scale metabolic model, metabolic simulation, dynamic flux balance analysis, data approximation

## Abstract

Flux balance analysis (FBA) is used to improve the microbial production of useful compounds. However, a large gap often exists between the FBA solution and the experimental yield, because of growth and byproducts. FBA has been extended to dynamic FBA (dFBA), which is applicable to time-varying processes, such as batch or fed-batch cultures, and has significantly contributed to metabolic and cultural engineering applications. On the other hand, the performance of the experimental strains has not been fully evaluated. In this study, we applied dFBA to the production of shikimic acid from glucose in *Escherichia coli*, to evaluate the production performance of the strain as a case study. The experimental data of glucose consumption and cell growth were used as FBA constraints. Bi-level FBA optimization with maximized growth and shikimic acid production were the objective functions. Results suggest that the shikimic acid concentration in the high-shikimic-acid-producing strain constructed in the experiment reached up to 84% of the maximum value by simulation. Thus, this method can be used to evaluate the performance of strains and estimate the milestones of strain improvement.

## 1. Introduction

The microbial production of various useful compounds has been actively studied. With the recent development of synthetic biotechnology, the production of useful compounds that microorganisms do not naturally produce can be made possible by introducing biosynthetic pathways (synthetic metabolic pathways), designed by combining heterologous and modified (mutant) enzymes. In addition, active research on the use of metabolic models has contributed to an effective improvement in the yield of useful compounds [1,2,3,4]. In such studies that often involve a genome-scale metabolic model (GSM), Flux Balance Analysis (FBA) is frequently used for metabolic simulations [5].

FBA is a method that assumes a steady state in which the concentration of a metabolite does not change with time. An FBA solution (the distribution of metabolic fluxes) that maximizes or minimizes the objective function is searched, using a stoichiometric matrix composed of stoichiometric coefficients of reactions that constitute the metabolic model [6]. In the FBA-based method, a simulation can be performed relatively easily, using a large-scale GSM, and the analyses have provided various metabolic engineering strategies for the improved production of useful compounds. FBA is also used for analyzing the steady state obtained by continuous culture and the snapshot of a time-varying system [7].

In addition, the theoretical maximum yield under given constraints (conditions) can be obtained with FBA, although this is usually different from the actual yield in a dynamic production process with growth. From this point of view, dynamic FBA (dFBA) has been developed, which is an extension of FBA applicable to dynamic production processes, such as batch and fed-batch cultures with growth [8]. The dFBA method expresses time-dependent changes in the substrates, the products, oxygen, and cell concentrations in the culture medium by ordinary differential equations (ODEs), and expresses the intracellular metabolic reactions by GSM for FBA.

The dynamic FBA function in the COBRA Toolbox implements the method of Mahadevan et al. [9], DyMMM [10] and DFBAlab [11]. A plurality of kinetic parameters is included in dFBA, to express the substrate/oxygen concentration by the differential equations in the medium. Therefore, it is necessary to estimate values of the arameters to reproduce experimental time-course data. For parameter estimation, manual tuning [12], and the nonlinear least squares method are used. The dFBA method has been used for the optimization of substrate feeding profiles and simulation in ethanol production by fed-batch cultures of yeast [13], and for the co-culture of yeasts *Saccharomyces cerevisiae* and *Scheffersomyces stipitis* [14], as well as *S. cerevisiae* and *Escherichia coli* [12]. In these studies, dFBA was performed, based on the cultural time-course data of each bacterium, and the parameters for reproducing the experimental data were estimated. After that, dFBA simulation of co-culture was carried out to inform culture engineering strategies, such as initial cell ratios and aeration conditions [12,14]. Furthermore, dFBA has been extended to integrated dFBA (idFBA) by the integration of signaling, metabolic, and regulatory networks [15], and to integrated FBA (iFBA) by the integration of ODEs and regulatory Boolean logic [16] for more detailed analyses. Another example of dFBA using time-course data is the improved production of valuable compounds by green algae [17].

Thus, dFBA has made various contributions to non-steady-state systems, such as batch and fed-batch cultures. Moreover, since dFBA is applied to systems involving cell growth, such as batch and fed-batch cultures, the theoretical maximum production concentrations and yields can be estimated under conditions closer to the production process than FBA. By comparing these maximum production concentrations and yields with the experimental values, it was also possible to estimate the differences between the simulated maximum values and the experimental results, and to evaluate production performance in the target compound production of the strain, which could provide useful information as to whether there is room for improvement in the production of the target compound. However, to our knowledge, although seen as part of the lexicographic optimization in DFBAlab [11], simulation and analysis are rarely performed in non-steady-state systems with the objective function of maximizing the production of the target substance like FBA. In this study, therefore, we applied dFBA to estimate the difference between the simulated maximum production concentration of the target compound and the experimental value under the same constraints, such as substrate consumption and cell growth, and to evaluate production performance in the experimental strain. In the dFBA part of this study, instead of estimating the kinetic parameters of the differential equations, the approximate values of the time-course data were converted and used in FBA constraints. In the FBA part, we performed a two-step optimization with different objective functions. The obtained fluxes for substrates, growth, and products were converted into concentrations by numerical integration and used to estimate the difference between the simulated maximum production concentration of the target compound and the experimental values, and for the evaluation of production performance in the experimental strain.

**Figure 1:**
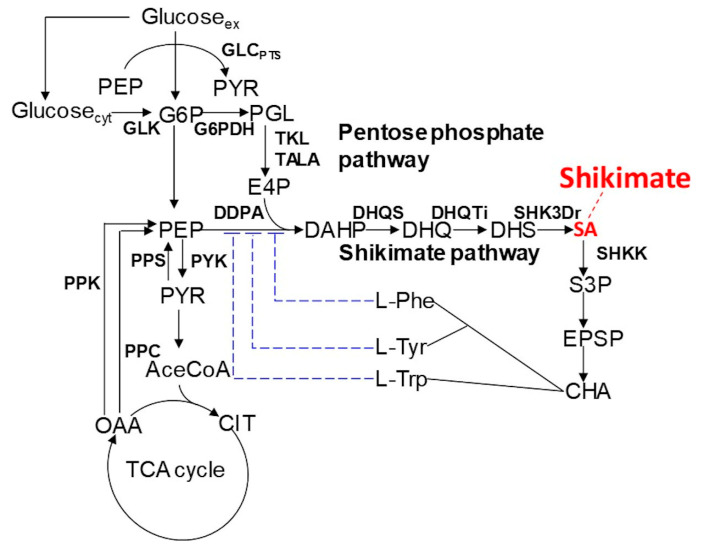
Schematic diagram of the metabolic pathway from glucose to shikimic acid in *E. coli*. Arrows indicate metabolic reactions, and hammerhead arrows indicate feedback inhibition.

In this study, we applied dFBA to shikimic acid production in *E. coli* (Figure 1) as a case study and verified its effectiveness. *E. coli* was selected as the host, because there is considerable knowledge of this model, it has been actively used and researched as a production host, and there are many studies on metabolic simulations using it as the metabolic model [18,19,20,21,22,23]. In addition, shikimic acid was selected as the target compound because shikimic acid is a hub compound with known effects of its derivation on various useful compounds such as alkaloids, opioid compounds and other aromatic compounds. Improved production of shikimic acid is therefore desirable [24,25]. Furthermore, many genetic modifications have been made [26].

In this study, dFBA, which repeats FBA sequentially without estimating kinetic parameters in differential equations, was applied to the production of shikimic acid in *E. coli* using polynomial approximation of the time-course data of the experiment. The results suggested that the shikimic acid production concentration of the experimental strain was about 84% of the theoretical value under the same constraints of substrate consumption and bacterial growth. Thus, this method could be an indicator of the attainment of the productivity of experimental strains in the production of useful compounds.

## 2. Results

### 2.1. Extraction and Approximation of Time-Course Data in Literature

**Figure 2:**
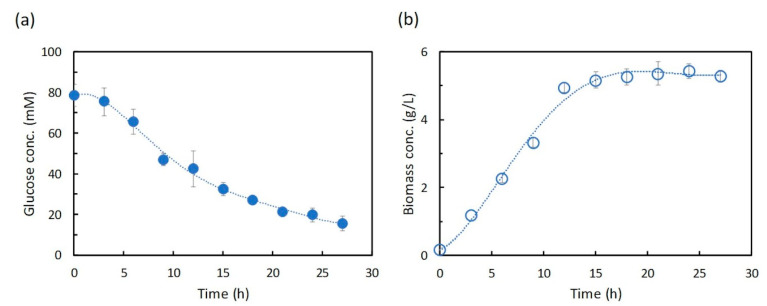
Approximated results of glucose (**a**) and biomass (**b**) concentrations. The symbols indicate measured values extracted from a previous publication by Chen et al. [27], and the lines indicate the results of polynomial approximations.

The shikimic acid production from glucose by *E. coli* by Chen et al. [27] was used in this study. Figure 3 of Chen et al. [27] shows time-course data for glucose and biomass, but not time-course data for shikimic acid. Therefore, numerical data were manually extracted from the time-course data of glucose and biomass concentrations and the bar charts of shikimic acid in the cited figure in [27] using WebPlotDigitizer [28] (Supplementary Table S1). The extracted time-course data on glucose and biomass concentrations in SA5/pTH-aroG^fbr^-ppsA-tktA were approximated by polynomial regression (the fifth order) using the least squares method to obtain Equations (1) and (2). As shown in Figure 2, the results of the implemented polynomial approximation successfully reproduced the experimental data extracted from the cited reference.

Approximate equation for glucose concentration

(1) $$Glt\left(t\right)=4.24753\times 10^{-5}t^{5}-3.43279\times 10^{-3}t^{4}+1.01057\times 10^{-1}t^{3}-1.21840t^{2}+1.89582t+7.85035\times 10$$

Approximate equation of bacterial cell (biomass) concentration

(2) $$\begin{array}{l} X\left(t\right)=-1.51269\times 10^{-6}t^{5}+1.56060\times 10^{-4}t^{4}-5.42057\times 10^{-3}t^{3}+6.43382\times 10^{-2}t^{2}+1.37275\times 10^{-1}t\\ +1.73785\times 10^{-1} \end{array}$$

where, *t* indicates an arbitrary time (h), and Glc (*t*) and *X*(*t*) indicate approximate equations of concentrations of glucose and biomass, respectively.

### 2.2. Preparing Constraints for Dynamic Flux Balance Analysis

**Figure 3:**
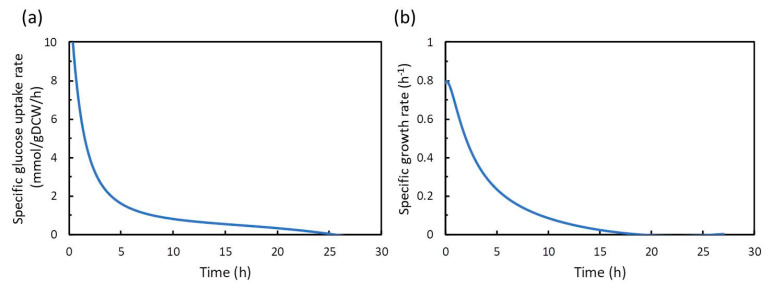
Time-course data of the approximated specific glucose uptake rate (**a**) and the approximated specific growth rate of cells (biomass) (**b**) estimated from the approximate formula.

In the dFBA of this study, the FBA is sequentially performed at an arbitrary time. Therefore, it is necessary to prepare a time course of the specific uptake rate of the substrate (glucose), and the specific growth rate of the bacterial cell (biomass) as constrains (lower and upper boundaries) in dFBA. The constraints to dFBA are the values of the flux at any time *t* (h) [mmol/g dry cell weight (DCW)/h; cell growth (biomass synthesis) is exceptionally h^−1^]. On the other hand, the units of the data extracted from the experimental data and their approximated values are mM or g/L. Therefore, it is necessary to perform unit conversions so that the values can be used as constrains in dFBA. The equations of glucose and cell concentrations were polynomially approximated as a function of time t. These equations were differentiated with respect to t, and then divided by the equation of cell concentrations, to obtain the equations of specific glucose uptake and growth rate. The approximated time course of the obtained specific glucose uptake rate and specific growth rate is shown in Figure 3, and the approximate equations are as follows.

The approximated specific glucose uptake rate $v_{uptake\_Glc}^{approx}\left(t\right)$ (mmol/g DCW/h) =

(3) $$\left(-2.123765\times 10^{-4}t^{4}+1.373116\times 10^{-2}t^{3}–3.03171\times 10^{-1}t^{2}+2.4368\times t–1.89582\right)/X\left(t\right)$$

The approximated specifics growth rate $\mu^{approx}\left(t\right)$ (h^−1^) =

(4) $$\left(-7.56345\times 10^{-6}t^{4}+6.24240\times 10^{-4}t^{3}–1.62617\times 10^{-2}t^{2}+0.128676t+0.137275\right)/X\left(t\right)$$

### 2.3. Dynamic Flux Balance Analysis

**Figure 4:**
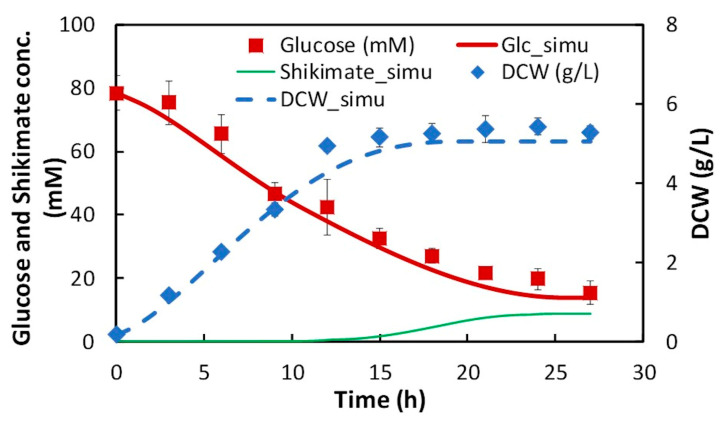
Comparison of time-course data on concentrations of glucose, dry cell weight (DCW), and shikimic acid obtained by dynamic flux balance analysis (FBA) (dFBA) to experimental values. Symbols (square: glucose, rhombus: dry cell weight) and lines (bold: glucose, dashed: DCW, solid: shikimic acid) indicate the values obtained by the experiment and simulation, respectively.

In the dFBA, rates of specific glucose uptake and specific growth based on the approximated glucose and cell concentrations were sequentially used as the constraints of the FBA. The GSM of *E. coli* used for FBA was iJO1366 [29]. The objective functions in the FBA part were maximized growth (the first step) or shikimic acid production flux (the second step), respectively. It was assumed that there was no shikimic acid production during the induction phase at the beginning of the culture, and the period was designated as 0 to 2 h. In the first step of FBA, if the valid solution cannot be obtained due to the constraints used (solver status was infeasible) at time t, then FBA was repeated until the valid solution was obtained (solver status was optimal solution), while relaxing constraints on the specific rates of glucose uptake and growth by changing the lower and upper boundaries of those fluxes, which are shown in Equations (5) and (6).

(5) $$v_{uptake\_Glc}^{approx}\left(t\right)\times \left\{1-0.01\times \left(n-1\right)\right\}\leq v_{uptake\_Glc}\leq v_{uptake\_Glc}^{approx}\left(t\right)\times \left\{1+0.01\times \left(n-1\right)\right\}$$

(6) $$\mu^{approx}\left(t\right)\times \left\{1-0.01\times \left(n-1\right)\right\}\leq \mu \leq \mu^{approx}\left(t\right)\times \left\{1+0.01\times \left(n-1\right)\right\}$$

where, $v_{uptake\_Glc}^{approx}\left(t\right)$ is the specific glucose uptake rate at time t obtained by Equation (3), $v_{uptake\_Glc}$ is the specific glucose uptake rate at time t by the first step of FBA, $\mu^{approx}\left(t\right)$ is the specific growth rate at time t obtained by Equation (4), μ is the specific growth rate at time t by the first step of FBA, and n is the n^th^ FBA repetition. Thus, the time course of each flux was obtained. Among the obtained fluxes, glucose uptake, cell growth (biomass synthesis), and shikimic acid production (discharge) fluxes were numerically integrated, and time-course data of each concentration were obtained (Figure 4), which demonstrated that the time-course data obtained by the dFBA could accurately reproduce the experimental data (Figure 4).

**Figure 5:**
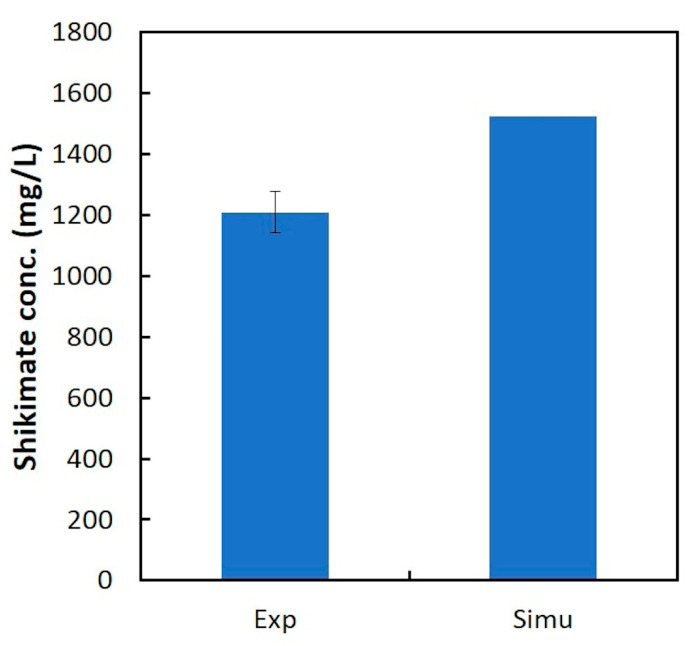
Comparison of shikimic acid concentration obtained by dFBA at 27 h to the experimental value.

On the other hand, the final shikimic acid concentration obtained by the experiment was 75–84% of that obtained by dFBA (Figure 5), suggesting that there is a little room for improvement in shikimic acid production under the same conditions of substrate consumption and growth.

## 3. Discussion

In this study, we applied dFBA for estimating the difference between the simulated maximum concentration and the experimental value of the target compound, and for evaluating the production performance of the experimental strain under the same constraints of substrate consumption and cell growth as those in the experiment. The dFBA method is used to estimate and obtain the time courses of metabolic fluxes in the metabolic model by repeatedly performing FBA at each time using the time-course data of the substrate, cell growth, and product concentrations from the experiment. As a case study, we applied dFBA to shikimic acid production from glucose by *E. coli* to verify its usefulness in microbial production of useful compounds.

In this study, dFBA was the method for obtaining a set of time courses of metabolic fluxes using data polynomial approximation. In addition, in two-step FBA, the specific rates of the substrate uptake and the growth of bacterial cells are used as constraints in first step FBA, and the maximization of the production flux of the target compound is used as the objective function in the second FBA. As a result, the difference between the simulated maximum concentration and the experimental value of the target production can be estimated. Furthermore, the production performance of the target compound in the experimental strain can be compared to the theoretical maximum value obtained by dFBA, which provides useful information on the production of compounds by microorganisms.

In this study, the FBA is repeatedly performed at each time point. However, unlike basic dFBA, the dFBA in this study uses a polynomial approximation of the time-course data obtained from the experiment. There is no need to estimate the parameters in the differential equations. Therefore, the time courses of the metabolic fluxes in the metabolic model can be acquired more easily. Further, as in the case of basic dFBA, when a sudden change in the metabolic fluxes, such as a phase shift, is observed in the time course of the obtained metabolic flux, information on dynamic control, such as switching, can be provided by dFBA. In addition, the flux of oxygen uptake can be used as culture engineering strategies, such as the optimization of aeration conditions. However, unlike basic dFBA, the ODE part composed of differential equations is not formulated in the Michaelis-Menten or Monod type, and those kinetic parameters are not estimated, so it is difficult to use it to simulate co-culture performed in the previous publication [13,14]. Also, unlike basic dFBA, it is not possible to simulate when changing the parameters of substrate and oxygen uptake. Another technique similar to dFBA in this study using polynomial approximation of experimental time-course data is the dynamic metabolic flux analysis (DMFA) [30,31]. Compared to dFBA, the purpose of using data approximation and interpolation is the same, but the linear approximation and B-spline are used in DMFA. Furthermore, metabolic flux analysis is used instead of FBA in metabolic simulations. In this study, polynomial approximation was used; but in DMFA, changing linear interpolation to B-spline improved the results [31]. Therefore, depending on the data to be approximated or interpolated, the results of the approximation or interpolation are expected to be improved by using a different method, and the effect can be expanded to the results of dFBA.

Since the dFBA, in this study, performs the simulation using the polynomial approximation of the experimental time-course data, there is a drawback, in that the simulation cannot proceed unless the approximation is successful. In addition, if the metabolic model used for the simulation is not accurate, the experimental results cannot be reproduced. The dFBA simulated dynamic systems that change with time, such as batch or fed-batch cultures. However, unlike the simulation using a dynamic (kinetic) model, FBA assuming a steady state is used for the intracellular simulation. Therefore, it is impossible to express the limitation of metabolic flux or the accumulation of intermediate metabolites, due to some factors such as imbalance of enzyme amounts and cofactor supply, feedback regulations.

Therefore, in this study, the dFBA can more easily acquire the time courses of the metabolic fluxes in the metabolic model using time-course data approximation. Additionally, in compound production, by using the fluxes of specific substrate uptake and growth obtained by approximation as FBA constraints, it is possible to estimate the difference between the current strain and the theoretical maximum values, and to estimate the room of improvement.

In a more detailed analysis, such as pathway optimization, dFBA does not reach simulation and analysis using a dynamic (kinetic) model. In the construction of a dynamic model, parameter estimation is a very costly operation, which is a barrier to the construction of a large-scale dynamic model. It has been suggested that combining flux time-course data with metabolome-based intracellular metabolite concentration time-course data facilitates parameter estimation [32,33,34,35,36,37]. Therefore, estimation of the time courses of metabolic fluxes using dFBA is expected to reduce the burden of constructing a dynamic model.

## 4. Materials and Methods

**Figure 6:**
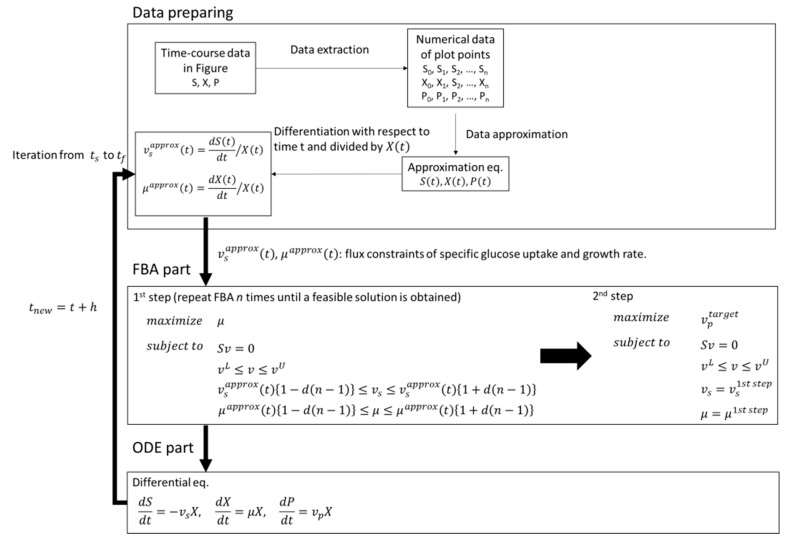
Workflow of methodology from time-course data in figure to dynamic flux balance analysis. Under the section Data preparing, S, X, and P indicate substrate, cell, and product, respectively. Subscripts (0, 1, …, n) show sampling points. *S(t)*, *X(t)*, and *P(t)* represent approximate equation of substrate, cell, and product, respectively. *v_s_*^approx^*(t)*_,_ *μ^approx^(t)* represent the approximated specific substrate uptake and growth rates, respectively. *t_s_, t_f_*, and *h* indicate start time, finish time, and time step for calculation, respectively. In the section FBA, *S*: stoichiometric matrix; *v*: flux vector; *d*: the arbitrary constant for changing boundary; *n*: *n*^th^ repetition; superscript *L*: lower boundary; *U*: upper boundary; target: target product.

The workflow of the method is summarized in Figure 6.

### 4.1. Creating A Dataset

#### 4.1.1. Data Extraction

Using the online version of WebPlotDigitizer 4.2 (https://automeris.io/WebPlotDigitizer/) [28], the plot points were specified manually and numerical data was extracted from the time-course data of substrates and cell concentrations in the figure of the literature [27].

#### 4.1.2. Data Conversion and Approximation

The cell concentration obtained from the literature is either DCW or OD values. In this study, the conversion factor of DCW per OD in *E. coli* is 0.33 g DCW/OD_600_ [38]. If the unit of the glucose concentration was g/L, it was converted to mM.

The extracted time-course numerical data was divided into arbitrary sections as needed, and a polynomial approximation using the least squares method was performed. The approximated concentrations glucose and cells were expressed as a function of time *t* by the following polynomial equations (Equations (7) and (8)).

(7) $$Glc\left(t\right)=a_{k}t^{k}+a_{k-1}t^{k-1}+\cdots a_{1}t+a_{0}+\varepsilon_{Glc}$$

(8) $$X\left(t\right)=b_{k}t^{k}+b_{k-1}t^{k-1}+\cdots b_{1}t+b_{0}+\varepsilon_{X}$$

where, $a_{i},b_{i}\left(i=0,1,2,\cdots ,k\right)$ are polynomial coefficients of approximated glucose and cell concentration; k is order; t is time; and $\varepsilon_{Glc},\varepsilon_{X}$ are errors in polynomial approximation of glucose and cell concentration, respectively. The obtained approximation results were compared to numerical data extracted from the figure and evaluated.

The specific rate of glucose uptake (mmol/g/h) and the specific rate of growth (h^−1^) used for the dFBA constraints were calculated by dividing the derivatives of the approximation equations of the substrate and cell concentration with respect to the time *t* by the cell concentration (g/L) at that time.

### 4.2. Dynamic Flux Balance Analysis

In the dFBA method, instead of formulating a differential equation and estimating the kinetic parameter values in the equations, the obtained values by approximation equations are used in the FBA at any time *t* (h).

The specific substrate uptake rate and the specific growth rate given as input constraints to the FBA part were calculated by differentiating the approximate equation of the substrate concentration and the bacterial cell concentration divided by the approximate expression of the bacterial cell concentration at time *t*. Depending on the approximation result, the specific glucose uptake rate and the specific growth rate may show negative values. In such cases, 0 was compulsorily set. In addition, as mentioned in Section 2.3, there are cases where a valid solution cannot be obtained (solver status is infeasible) by the first step FBA with the values of the specific substrate uptake rate and the specific growth rate given as constraints. Therefore, the flux distribution at each time was acquired by repeating the first step FBA process until a valid solution was obtained (solver status was optimal solution), while relaxing constraints on the specific rates of glucose uptake and growth by changing the lower and upper boundaries of those fluxes (first step FBA at FBA part in Figure 6). In this study, d was set 0.01.

As mentioned above, the FBA part is divided into two steps. In the first step, the objective function was to maximize the specific growth rate while relaxing constraints as needed. In the second step, the specific growth rate was fixed at the value obtained in the first step, and the production flux of the target compound was maximized as an objective function. The step size of the calculation was 0.01 (h), and FBA at any time *t* (h) was sequentially performed to obtain time-course data of the flux. Using this method, the time-course data of metabolic fluxes were collected.

The time course of extracellular metabolites and the cell concentration were calculated by numerically integrating the time courses of the fluxes obtained by dFBA using Simpson’s rule. In addition, the final concentration of the target compound obtained by numerical integration was regarded as the maximum production concentration of the target compound under experimental substrate consumption and growth constraints. The obtained maximum production concentration of the target compound was compared to the actual experimental data, and the production performance of the strain used in the experiment under defined constraints and its theoretical maximum value were estimated.

The MATLAB© 2018b (The MathWorks Inc. Natick, MA, USA) and COBRA Toolbox (https://github.com/opencobra/cobratoolbox.git) were used for the dFBA simulation, and Gurobi 8.1 (Gurobi Optimization, LLC) was used for the FBA solver. The home-made MATLAB script (computer code) used for the calculation of time-course of metabolic fluxes are available on GitHub (https://github.com/yukuriya3/Calculation_for_Ecoli_skm_pro).

### 4.3. Case Study: Shikimate Production from Glucose by E. coli

The above dFBA was applied to the example of shikimic acid production from glucose in *E. coli*. Time-course data on shikimate production by *E. coli* was selected from previous publication [27]. The numerical time-course data on shikimic acid production were extracted from the referenced figure manually using WebPlotDigitizer 4.2 (https://automeris.io/WebPlotDigitizer/) [28]. The extracted time course data (Supplementary Table S1) was divided into sections as needed, and polynomial approximation was performed. Depending on the unit of the cell concentrations, it is necessary to obtain a conversion coefficient for the DCW (g) or OD_600_ value from the literature for the conversion. In this case, the unit of cell concentration was (g/L) for DCW in the time course data, which was used as is.

The values for FBA constraints in dFBA were converted to specific glucose uptake rate (mmol/g DCW/h) and specific growth rate (h^−1^) by dividing the derivatives of the approximation equations for glucose and cell concentration with respect to time *t* by the corresponding cell concentration (g/L).

In the study by Chen et al., the medium contained amino acids and the concentrations of L-phenylalanine L-tyrosine, and L-tryptophan were 0.7 g/L, 0.7 g/L, and 0.35 g/L, respectively [27]. Therefore, the flux constraints were changed to accommodate these amino acids in the metabolic model. In addition, based on the integrated values from the FBA result, the uptake of each amino acid was set to stop when the concentrations of these amino acids becomes 0. Using the constraints for FBA obtained at each time *t*, the FBA was sequentially repeated to obtain time-course data of the flux.

The concentrations of extracellular substrates (glucose, amino acid), shikimic acid, and cells were calculated by numerical integration using the Simpson’s rule, based on the output values sequentially obtained FBA. From these results, data at the time of maximum production of the target compound under the constraints of experimental substrate consumption and cell growth were estimated.

By comparing with the actual experimental data, the theoretical yield using the current strain was estimated under the same constraints on substrate consumption and bacterial growth as in the experiment. The actual yield of the strains compared to the theoretical yield under the experimental constraints will inform whether the yield will be increased by optimizing the culture process, or by metabolic engineering in the future. These results will also be used for strain evaluation and planning of engineering strategies.

## Supplementary Materials

The following are available online at https://www.mdpi.com/2218-1989/10/5/198/s1, Table S1: Numerical data extracted from data of *E. coli* SA5/pTH-aroG^fbr^-ppsA-tktA in Figure 3 of Chen et al., 2014 [27].
