# Supplementary material for: Dynamic Flux Balance Analysis to Evaluate the Strain Production Performance on Shikimic Acid Production in Escherichia coli
Source: Metabolites. 2020 May 15;10(5):198. doi: 10.3390/metabo10050198 (PMC7281464; doi:10.3390/metabo10050198)
Supplement: Supplementary file 1 [file metabolites-10-00198-s001.pdf]

Supplementary materials

Table S1 Numerical data extracted from data of *E. coli* SA5/pTH-aroG^{fbr}-ppsA-tktA in Fig. 3 of Chen *et al.*, 2014 (ref. [24] in article)

Time (h)	Glucose (g/L)	Biomass (g/L)	Shikimic acid (mg/L)
0	14.14	0.17	-
3	13.61	1.18	-
6	11.80	2.26	-
9	8.43	3.33	-
12	7.66	4.94	-
15	5.84	5.16	-
18	4.85	5.26	-
21	3.87	5.36	-
24	3.55	5.43	-
27	2.78	5.29	1209
